# Retraction of: Identification of biological markers for better characterization of older subjects with physical frailty and sarcopenia

**DOI:** 10.1515/tnsci-2020-0142

**Published:** 2020-09-11

**Authors:** Bertrand Fougère, Bruno Vellas, Gabor Abellan van Kan, Matteo Cesari

**Affiliations:** Gérontopôle, Centre Hospitalier Universitaire de Toulouse, Toulouse, France; Inserm UMR1027, Université de Toulouse III Paul Sabatier, Toulouse, France

The lead author and the publisher would like to inform that the article “Identification of biological markers for better characterization of older subjects with physical frailty and sarcopenia” (https://doi.org/10.1515/tnsci-2015-0009) has been retracted as per their request.

The article bears the hallmarks of plagiarism, using the unreferenced quotes from, among others, the following papers:

1. Ali S, Garcia JM. Sarcopenia, cachexia and aging: diagnosis, mechanisms and therapeutic options – a mini-review. Gerontology. 2014;60(4):294–305.
2. Bhasin S, He EJ, Kawakubo M, Schroeder ET, Yarasheski K, Opiteck G, et al. N-terminal propeptide of type III procollagen as a biomarker of anabolic response to recombinant human GH and testosterone. J Clin Endocrinol Metab. 2009;94(11):4224–33. doi: 10.1210/jc.2009-1434.
3. Ceglia L. Vitamin D and skeletal muscle tissue and function. Mol Aspects Med. 2008 Dec;29(6):407–14.
4. Cesari M, Fielding RA, Pahor M, Goodpaster B, Hellerstein M, van Kan GA, et al. International Working Group on Sarcopenia. Biomarkers of sarcopenia in clinical trials-recommendations from the International Working Group on Sarcopenia. J Cachexia Sarcopenia Muscle. 2012;3(3):181–90. doi: 10.1007/s13539-012-0078-2.
5. Cooper C, Dere W, Evans W, Kanis JA, Rizzoli R, Sayer AA, et al. Frailty and sarcopenia: definitions and outcome parameters. Osteoporosis Int. 2012;23:1839–48. doi: 10.1007/s00198-012-1913-1.
6. Cruz-Jentoft AJ, Michel JP, Cruz-Jentoft AJ, Michel J-P. Sarcopenia: a useful paradigm for physical frailty. Eur Geriatr Med. 2013;4(2):102–5.
7. Drey M, Sieber CC, Bauer JM, Uter W, Dahinden P, Fariello RG, et al. C-terminal agrin fragment as a potential marker for sarcopenia caused by degeneration of the neuromuscular junction. Exp Gerontol. 2013;48(1):76–80. doi: 10.1016/j.exger.2012.05.021.
8. Feng J, He W, Song Y, Wang Y, Simpson R, Zhang X, et al. Platelet-derived growth factor receptor beta: a novel urinary biomarker for recurrence of non-muscle-invasive bladder cancer. PLoS One. 2014;9(5):e96671. doi: 10.1371/journal.pone.0096671.
9. Fulle S, Protasi F, Di Tano G, Pietrangelo T, Beltramin A, Boncompagni S, et al. The contribution of reactive oxygen species to sarcopenia and muscle ageing. Exp Gerontol. 2004;39(1):17–24. doi: 10.1016/j.exger.2003.09.012.
10. Patel SS, Molnar MZ, Tayek JA, Ix JH, Noori N, Benner D, et al. Serum creatinine as a marker of muscle mass in chronic kidney disease: results of a cross-sectional study and review of literature. J Cachexia Sarcopenia Muscle. 2013;4(1):19–29. doi: 10.1007/s13539-012-0079-1.
11. Scharf G, Heineke J. Finding good biomarkers for sarcopenia. J Cachexia Sarcopenia Muscle. 2012 Sep;3(3):145–8.

